# Anterior cingulate cortex, but not amygdala, modulates the anxiogenesis induced by living with conspecifics subjected to chronic restraint stress in male mice

**DOI:** 10.3389/fnbeh.2022.1077368

**Published:** 2023-01-06

**Authors:** Lara Maria Silveira, Ligia Renata Rodrigues Tavares, Daniela Baptista-de-Souza, Isabela Miranda Carmona, Paulo Eduardo Carneiro de Oliveira, Ricardo Luiz Nunes-de-Souza, Azair Canto-de-Souza

**Affiliations:** ^1^Psychobiology Group, Department of Psychology/Centro de Educação e Ciências Humanas (CECH), Universidade Federal de São Carlos, São Carlos, São Paulo, Brazil; ^2^Graduate Program in Psychology, Centro de Educação e Ciências Humanas (CECH)-Universidade Federal de São Carlos, São Paulo, Brazil; ^3^Joint Graduate Program in Physiological Sciences, Universidade Federal de São Carlos (UFSCar)/Universidade Estadual Paulista (UNESP), São Carlos, São Paulo, Brazil; ^4^Laboratory of Pharmacology, School of Pharmaceutical Sciences, Universidade Estadual Paulista (UNESP), Araraquara, São Paulo, Brazil; ^5^Institute of Neuroscience and Behaviour, Ribeirão Preto, São Paulo, Brazil

**Keywords:** empathy, chronic restraint stress, anxiety, anterior cingulate cortex, amygdala, mice

## Abstract

Cohabitation with a partner undergoing chronic restraint stress (CRE) induces anxiogenic-like behaviors through emotional contagion. We hypothesized that the anterior cingulate cortex (ACC) and the amygdala would be involved in the modulation of this emotional process. This study investigated the role of the ACC and amygdala in empathy-like behavior (e.g., anxiety-like responses) induced by living with a mouse subjected to CRE. Male Swiss mice were housed in pairs for 14 days and then allocated into two groups: cagemate stress (one animal of the pair was subjected to 14 days of restraint stress) and cagemate control (no animal experienced stress). Twenty-four hours after the last stress session, cagemates had their brains removed for recording FosB labeling in the ACC and amygdala (Exp.1). In experiments 2 and 3, 24 h after the last stress session, the cagemates received 0.1 μL of saline or cobalt chloride (CoCl_2_ 1 mM) into the ACC or amygdala, and then exposed to the elevated plus-maze (EPM) for recording anxiety. Results showed a decrease of FosB labeling in the ACC without changing immunofluorescence in the amygdala of stress cagemate mice. Cohabitation with mice subjected to CRE provoked anxiogenic-like behaviors. Local inactivation of ACC (but not the amygdala) reversed the anxiogenic-like effects induced by cohabitation with a partner undergoing CRE. These results suggest the involvement of ACC, but not the amygdala, in anxiety induced by emotional contagion.

## Introduction

Stress is an omnipresent life experience that influences organisms’ daily behaviors and social functions. Many factors affect the pattern and magnitude of the stress response, for example, the type of stressor (Joëls and Baram, 2009). In addition, data from the literature suggest that emotional/psychological stress can play a critical role in the etiology of mood-related psychopathology, leading to a physiological and psychological imbalance of the organism, associated with several pathologies, as well as anxiety and depression (Wood, 2014). Furthermore, clinical and preclinical studies have indicated that anxiety-like behaviors can be indirectly induced in individuals who merely witness a stressful/traumatic event or are exposed to a distressed partner (Warren et al., 2013; Iñiguez et al., 2018; Karakilic et al., 2018).

In this sense, psychological stress promotes anxiogenesis both directly in an animal subjected to stress or socially transferred to the partner through emotional contagion, the simplest form of empathic demonstration (Chotiwat and Harris, 2006; Carneiro Paulo Eduardo Carneiro de Oliveira et al., 2017; Karakilic et al., 2018; Carneiro de Oliveira et al., 2022). In rodents, empathic behaviors are expressed by recognizing and reacting to the emotional state of their conspecifics (Langford et al., 2006; Martin et al., 2014; Lu et al., 2018). Overwhelming evidence suggests emotional contagion in rodents, yet the underlying neural mechanisms have not yet been fully elucidated (Panksepp, 2011; Gonzalez-Liencres et al., 2014; Mogil, 2015; Burkett et al., 2016; Keysers, 2022). Overall, to investigate the basis of empathy in animals, rodent models apply emotional stress or nociceptive stimuli to change behavioral responses in the observer partner (Baptista-de-Souza et al., 2015; Carneiro de Oliveira et al., 2017, 2022; Zaniboni et al., 2018; Benassi-Cezar et al., 2020; Tavares et al., 2021). For instance, besides inducing anxiety, nerve constriction, a procedure that produces neurophatic pain by itself (Benbouzid et al., 2008; Yalcin et al., 2014), also induced anxiogenic-like behaviors in mice living with a conspecific in chronic pain (Baptista-de-Souza et al., 2015; Carmona et al., 2016; Benassi-Cezar et al., 2020).

Animal models of stress have been used as a helpful tool to investigate the underlying mechanisms through which, stress exerts its detrimental effects on the functions of the brain and animal behavior (Blanchard et al., 2001; Mumtaz et al., 2018). In this sense, previous studies have shown that chronic restraint stress promotes anxiogenesis and pain hypersensitivity not only in the animal subjected to stress but also in the social partner, through emotional contagion, a type of empathic demonstration (Chotiwat and Harris, 2006; Carneiro de Oliveira et al., 2017). In the different developmental stages of life, exposure to chronic stress caused remarkable neuroplastic alterations related to area functions, and in the structure and function of receptors that affect synaptic neurotransmission (excitatory and inhibitory) in several regions of the brain (Salzberg et al., 2007; Mumtaz et al., 2018). Additionally, it is relevant to highlight that studies have focused on the development of the social brain beyond early childhood, and these studies support evidence from social psychology that adolescence represents a period of significant social development, besides the recognition of conspecifics and the understanding of others’ emotions (Blakemore, 2008; Atzil et al., 2018).

Brain areas that are involved in social cognitive processes and in understanding social emotions include the medial prefrontal cortex, the anterior cingulate cortex (ACC), the amygdala and the anterior insula. Bearing in mind these important findings, studies aiming to understand the biological basis of empathy-like responses indicated that the ACC and amygdala are promising brain areas involved in the modulation of responses induced by conspecific distress (Singer et al., 2004; Knapska et al., 2006; Carrillo et al., 2019; Benassi-Cezar et al., 2020; Keysers and Gazzola, 2021). For instance, it has been demonstrated that the painful stimulus is able to activate the ACC not only in animals that are suffering this aversive stimulus but also in their partners (Singer et al., 2004; Carrillo et al., 2019). Further, Burkett et al. (2016) demonstrated neuronal activation in the ACC of animals that observed their conspecifics subjected to a foot shock paradigm. Recently, our research group showed that the ACC inactivation (provoked by local injection of cobalt chloride–CoCl_2_) reversed the anxiogenic-like behavior induced by empathy for pain model (Benassi-Cezar et al., 2020). Beyond that, ACC inactivation impaired observational fear learning and reduced behavioral responses to pain in mice (Jeon et al., 2010). Regarding the role of the amygdala in empathy like-behavior, Jeon et al. (2010) demonstrated an increase in amygdala activation after observational fear learning provoked by observation of other mice receiving foot shocks.

Preclinical studies (Ramkumar et al., 2012; Tripathi et al., 2019) have shown that repeated restraint stress in rats induces anxiety-like behaviors concomitantly to volume decreasing and dendritic atrophy of ACC in clinical evidence (Asami et al., 2008; Greenberg et al., 2013). In contrast, Vyas et al. (2004) observed anxiogenic responses accompanied by spinogenesis and dendritic hypertrophy in the amygdala neurons of rats. In addition, hyperreactivity of the amygdala (Etkin et al., 2009) was described in patients with several anxiety disorders. Briefly, chronic stress differentially affects these brain areas, which points to ACC hypofunction and amygdala hyperactivity (Vyas et al., 2004; Ramkumar et al., 2012; Tripathi et al., 2019).

Even though the findings above have reported the involvement of the ACC and the amygdala in the modulation of emotional disorders (LeDoux, 2007; Lucassen et al., 2014) and emotional contagion for pain model (Benassi-Cezar et al., 2020; Tavares et al., 2021), very few is known about their roles in the nociceptive and behavioral responses exhibited by mice living with a conspecific subjected to chronic restraint stress. Here, we hypothesized that the increase of anxiogenic-like behavior induced by cohabiting with a conspecific in repeated stress is modulated by ACC and amygdala. Likewise, the CoCl_2_ inactivation of these structures would reverse the anxiety-induced by living with chronically stressed mice.

The present study investigated the involvement of the ACC and amygdala neurons in the expression of anxiety-like behaviors induced by emotional contagion in cagemates of animals experiencing stressful situations. To that end, three experiments were performed, namely (i) the quantification of FosB protein expression within the ACC and amygdala, (ii) the effects of chemical inhibition (through microinjection of CoCl_2_, a non-specific synaptic inhibitor) of the ACC and amygdala on the anxiety response of mice housed in pairs with a conspecific subjected to chronic restraint stress.

## Experimental procedures

### Subjects and ethics

Three hundred and sixteen male Swiss mice with 21-days-old (18–20 g) from the animal facility of the Federal University of São Carlos, São Paulo, Brazil, were used. The mice were housed two per cage [19 cm (width) × 30 cm (length) × 14 cm (height)] and were maintained under temperature and light controlled conditions (24 ± 1°C; 12-h light/dark cycle, lights on at 7:00 a.m.) with food and water *ad libitum*. The experiments were carried out during the light phase of the LD cycle (9:00 a.m.–4:00 p.m.) (Baptista-de-Souza et al., 2015; Zaniboni et al., 2018; Carneiro de Oliveira et al., 2022). Different batches of experimentally naïve mice were used for each of the three experiments and the postnatal day 21 (PND21; weaning), was used as the first day of the animals cohabitation (for details see Section “Experimental design”). All procedures were approved by the Federal University of São Carlos Ethics Committee on the Use of Animals (CEUA-UFSCar 7821030418) which complies with Brazilian and international guidelines for animal use and welfare.

### Restraint stress

Chronic restraint stress was performed with a PVC tube [14 cm (length) × 3 cm (diameter)]. One of the animals of each stress group was placed inside the tube 1 h a day for 14 consecutive days in its housing box in the presence of its conspecific (cagemate stress) in an adjacent room. The control and cagemate control were transferred to another adjacent room during the stress period for 1 h a day (Carneiro de Oliveira et al., 2017, 2022).

### Brain dissection

In experiment 1, cagemate control and cagemate stress were transcardially perfused with 30 mL of 1X phosphate-buffered saline (PBS) at pH 7.4, followed by 50 mL of 4% fresh paraformaldehyde (PFA). The brain was dissected and transferred to a 30% sucrose solution in PBS for 48 h at 4°C. The brains were then frozen, and three series of 35 μm thickness sections of the ACC and amygdala were cut in a cryostat at −20°C (Leica CM 1850) with the help of the Paxinos and Franklin atlas (Paxinos and Franklin, 2019). Section triplicates were placed in serial order in a 12-well plate containing 0.1 M phosphate buffer (PB) with 0.01% sodium azide and processed for immunofluorescence as described below.

### Immunofluorescence

Sections were washed three times in 0.1 M PB and then incubated in a blocking solution containing 10% Normal Goat Serum and 0.3% Triton-X 100 in 0.1 M PB for one h at room temperature with gentle rocking. Sections were incubated overnight with the primary antibody previously diluted in a blocking solution. After blocking, slices were incubated overnight with primary antibody (FosB, dilution 1:1000; Cat. N. AB184938, Abcam, USA). Sections were washed five times in 0.1 M PB and then incubated for 2 h at room temperature with biotinylated secondary anti-rabbit IgG antibody (Alexa-Fluor 488, dilution 1:1000; Abcam, USA). Following secondary incubation, sections were washed five times in 0.1 M PB, mounted onto glass slides, coverslipped using Fluoroshield Mounting Medium (Sigma-Aldrich, Brazil), and sealed with nail polish, once cured. The images from each slide were acquired using a fluorescence microscope (Axio Imager.D2, Carl Zeiss Microscopy, LLC, Thornwood-NY, USA) connected Zen Pro 2.0 software (Carl Zeiss Microscopy, LLC, Thornwood-NY, USA), and were analyzed using ImageJ software (NIH). Background staining was subtracted from each image before image analysis. The corrected total cellular fluorescence [CTCF = Integrated Density - (Area of selected tissue area × Mean fluorescence of background readings)] of the ACC and amygdala were measured by subtracting the background fluorescence from the integrated intensity and performed as described previously (McCloy et al., 2014; Baptista-de-Souza et al., 2020).

### Drugs

The drug microinjection into the ACC (experiment 2) or amygdala (experiment 3) was performed with cobalt chloride, 1.0 mM/0.1 μl (CoCl_2_; Sigma-Aldrich, Brazil). The drug was prepared in sterile saline (0.9% NaCl), which was used as control. The dose of CoCl_2_ was based on previous studies (Kretz, 1984; Benassi-Cezar et al., 2020; Tavares et al., 2021). The microinjections were performed bilaterally into the ACC or amygdaloid complex in a volume of 0.1 μl/side. The same saline volume was injected into the ACC and amygdala in the animals of the control group.

### Surgery and microinjection

In experiments 2 and 3, the cagemate control and cagemate stress were anesthetized with ketamine and xylazine [100 mg.kg^–1^ and 10 mg.kg^–1^ intraperitoneally (i.p.), respectively] and fixed in a stereotaxic frame (Insight Instruments, Brazil). Bilateral stainless-steel guide cannulae (25-gauge × 7 mm; Insight Instruments, Brazil) were then implanted and fixed to the skull using dental acrylic and jeweler’s screws. The bregma was considered the reference point, and the following coordinates were used to locate the target site in the ACC: anterior: + 1.0 mm; lateral: ± 0.2 mm; ventral: −1.3 mm; and in the amygdala: posterior: −0.8 mm; lateral: ±3.3 mm; ventral: −2.8 mm (Paxinos and Franklin, 2019). To reduce occlusion, each guide cannula was sealed with a stainless-steel wire to protect it from blockage at the time of surgery. At the end of the stereotaxic surgery, the animals received an intramuscular injection of the anti-inflammatory and analgesic drug ketoprofen (benzene acetic acid, 5 mg.kg^–1^) and an intramuscular injection of the antibiotic ceftriaxone (ceftriaxone sodium hemipentahydrate, 4 mg.kg^–1^) (Stepanovic-Petrovic et al., 2014). Subsequently, the mice were allowed to recover from the surgical procedure for 5 days. The cagemate control and cagemate stress received intra-ACC or intra-amygdala injection of saline or CoCl_2_ (1.0 mM/0.1 μl) 5 min before exposure to EPM. Solutions were injected using a microinjection unit (33-gauge stainless steel cannula; Insight Instruments, Brazil) that extended 2.0 mm beyond the tip of the bilateral guide cannulae in the amygdala and 0.5 mm beyond the tip of the guide cannulae in the ACC. The microinjection units were connected to a 10-μL Hamilton micro syringe through polyethylene tubing (PE-10). The flow rate was controlled with an infusion pump (BI 2000—Insight Instruments, Brazil) programmed to deliver 0.1 μL of each solution for 60 s. The microinjection procedure consisted of gently restraining the mice, inserting the injection unit, and infusing the solution for 60 s with an additional 90 s to maximize diffusion from the needle tip. The movement of a small air bubble in the PE-10 tube in microinjection confirmed the delivery of the solution (Nunes-de-Souza et al., 2000).

### Elevated plus-maze (EPM)

The EPM test was used to assess anxiety-like behavior. The apparatus was similar to that described by Lister (1987) and consisted of a wooden maze coated with plastic laminate, raised 38.5 cm from the floor, with four arms arranged in a plus format with two opposite arms closed by transparent glass walls (30 × 5 × 15 cm), connected by a common central platform (5 × 5 cm) with two opposite open arms (30 × 5 × 0.25 cm). All tests were conducted during the light phase of the light-dark cycle, under the illumination of 77 lux on the floor of the apparatus (Baptista-de-Souza et al., 2015). Animals were placed in the center of the maze facing an open arm. The number of entries and time spent in each arm were recorded for 5 min. An entry was considered when the animal put all four paws on an arm. Conventional measures were the percentage of open arm entries (%OE) [(open/total entries) × 100] and the percentage of time spent in open arms (%OT) [(time open/300) × 100]. These activities have been used as an index of anxiety behavior (Lister, 1987; Rodgers and Johnson, 1995). The number of closed-arm entries (CE) was used to measure locomotor activity in mice. Complementary behaviors measured were the percentage of time spent in the central platform [(central/total time) × 100], the number of head-dippings (exploratory movement of head/shoulders over sides of the maze), the percentage of protected head-dipping [(protected/total) × 100], the number of stretched attend postures (SAP; an exploratory posture in which the mouse stretches forward and retracts to the original position without locomotion), and the percentage of protected SAP [(protected/total) × 100]. Behaviors such as head-dipping and stretched attend postures were used to measure risk assessment (Treit et al., 1993; Cruz et al., 1994; Rodgers and Johnson, 1995). Besides, depending on where these behaviors were exhibited, they were counted as protected or unprotected. In line with previous studies, the closed arms and central platform were together designated as protected areas of the maze, while the open arms were designated as unprotected areas (Rodgers and Johnson, 1995). All sessions were recorded by a vertically mounted camera linked to a computer for posterior analysis. Test videos were scored by a highly trained observer using the free software package X-PloRat (Tejada et al., 2018).

### Body weight gain

To assess whether the restraint was effective, all subjects were weighted after the first and last stress sessions (15th and 28th day; see Section “Experimental procedures” for details). Weight gain was calculated based on the equation [(weight on 28th day) − (weight on 15th day)] (Chotiwat and Harris, 2006).

### Experimental design

**FIGURE 1:**
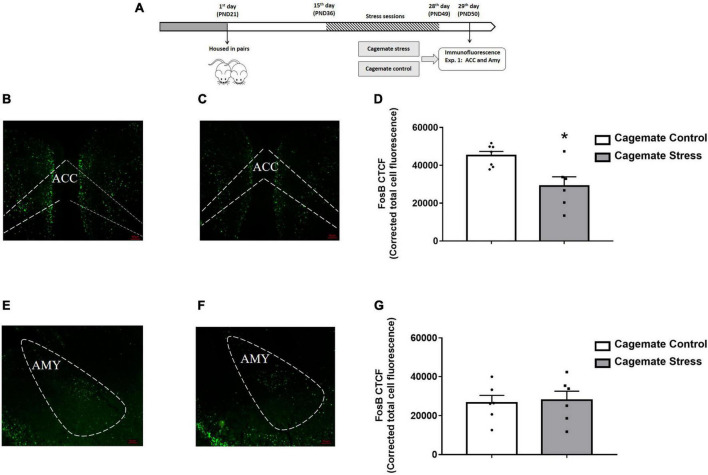
**(A)** Schematic representation of the experimental protocol (PND, postnatal day). **(B,C)** Representative photomicrographs of the anterior cingulate cortex (ACC) showing Fos-positive labeled cells in cagemate control and cagemates stress, respectively. **(D)** Number of Fos-positive labeled cells per corrected total cellular fluorescence of the ACC in cagemates that cohabited in pairs with mice subjected to chronic restraint stress compared to cagemate control. **(E,F)** Representative photomicrographs of the amygdala showing Fos-positive labeled cells in cagemates control and cagemates stress, respectively. **(G)** Number of Fos-positive labeled cells per corrected total cellular fluorescence of the amygdala in cagemates that cohabited in pairs with mice subjected to chronic restraint stress compared to cagemate control. All data are presented as mean ± SEM (*n* = 6–7). *p < 0.05 vs. respective cagemate control group. Student *t*-test. Amy, amygdala.

**FIGURE 2:**
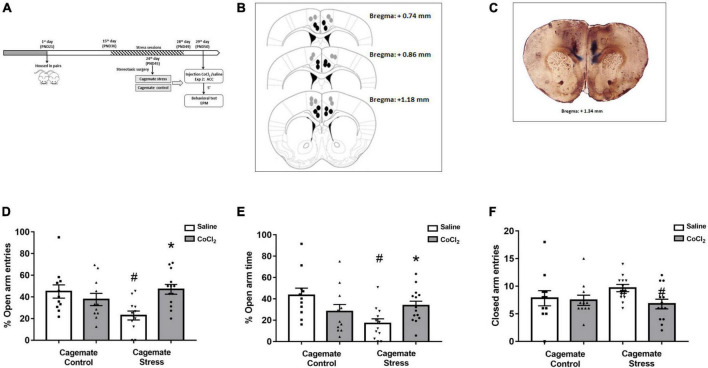
**(A)** Schematic representation of the experimental protocol (PND, postnatal day). **(B)** Schematic representation and **(C)** photomicrographs of histological results according to the Atlas of Paxinos and Franklin (2019) in the anterior cingulate cortex (ACC). The black circles represent the sites of drug infusion that were on-target within the ACC. The gray circles represent the locations in which the injection occurred outside the ACC. The number of dots in the panel is fewer than the number of animals used because of data overlapping (Bregma: +1.34 mm). **(D)** Effects of microinjection in the ACC of saline or CoCl_2_ (1 mM.100 nL^– 1^) in cagemates that cohabited in pairs with mice subjected to chronic restraint stress evaluated on the percentage of entry into the open arms in the EPM. **(E)** Effects of microinjection in the ACC of saline or CoCl_2_ (1 mM.100 nL^– 1^) in cagemates that cohabited in pairs with mice subjected to chronic restraint stress evaluated on the percentage of time in open arms in the EPM. **(F)** Effects of microinjection in the ACC of saline or CoCl_2_ (1 mM.100 nL^– 1^) in cagemates that cohabited in pairs with mice subjected to chronic restraint stress evaluated on the entry into the closed arms in the EPM. ACC, anterior cingulate cortex. All data are presented as mean ± SEM (*n* = 11–13). #*p* < 0.05 vs. respective cagemate control. *p < 0.05 vs. respective saline group. Two-way ANOVA, followed by Duncan *post-hoc* test.

**FIGURE 3:**
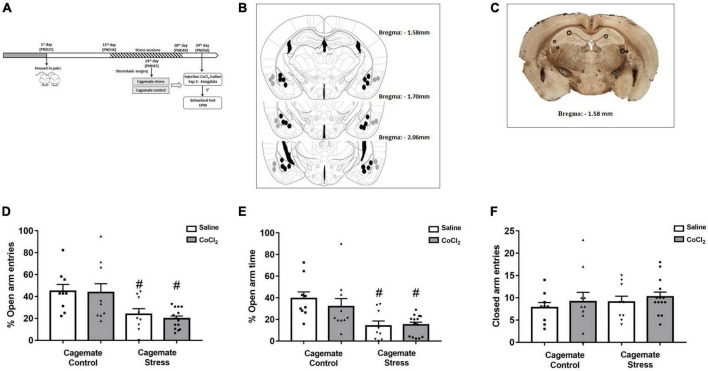
**(A)** Schematic representation of the experimental protocol (PND, postnatal day). **(B)** Schematic representation and **(C)** photomicrographs of histological results according to the Atlas of Paxinos and Franklin (2019) in the amygdala (Amy). The black circles represent the sites of drug infusion that were on-target within the amygdala. The gray circles represent the locations in which the injection occurred outside the amygdala. The number of dots in the panel is fewer than the number of animals used because of data overlapping (Bregma: –1.58 mm). **(D)** Effects of microinjection in amygdala of saline or CoCl_2_ (1 mM.100 nL^– 1^) in cagemates that cohabited in pairs with mice subjected to chronic restraint stress evaluated on the percentage of entry into the open arms in the EPM. **(E)** Effects of microinjection in amygdala of saline or CoCl_2_ (1 mM.100 nL^– 1^) in cagemates that cohabited in pairs with mice subjected to chronic restraint stress evaluated on the percentage of time in open arms in the EPM. **(F)** Effects of microinjection in amygdala of saline or CoCl_2_ (1 mM.100 nL^– 1^) in cagemates that cohabited in pairs with mice subjected to chronic restraint stress evaluated on the entry into the closed arms in the EPM. All data are presented as mean ± SEM (*n* = 9–14). #*p* < 0.05 vs. respective cagemate control. Two-way ANOVA, followed by Duncan *post-hoc* test.

On the 1st experiment day (1st day) or postnatal day 21 (PND21; weaning), mice were housed in pairs for 14 days and left undisturbed until the PND35 (14th day), except for cage cleaning. These 14 days before the beginning of stress sessions were applied for the establishment of familiarity between the pairs (Langford et al., 2006). On the 15th day (PND36) the animals were divided into two groups: stress, in which one animal of each pair was subjected to restraint stress for 1 h during 14 days until PND49 [the stress sessions occurred in the housing cage, in the presence of its conspecific] and control, in which no animal of the dyad was exposed to restraint stress. The observer conspecifics were denominated cagemate stress and cagemate control. In experiment 1, 24 h after the last stress session [29th day or PND50, mice were transcardially perfused and their brains were processed for the ACC and amygdala for immunofluorescence assay as previously described (Figure 1A)]. In experiments 2 and 3, on the 24th day (PND45), cagemate stress and cagemate control were subjected to stereotaxic surgery for implantation of bilateral guide cannulae intra-ACC or intra-amygdala at least 1 h after stress session and, after recovery, they returned to cohabit together. The stress sessions remained until the 28th day (PND49). Therefore, on the 29th day, cagemate stress and cagemate control received saline or CoCl_2_ microinjections into the ACC (Exp. 2) or into the amygdala (Exp. 3) and, after 5 min, were placed in the EPM to assess anxiety-like behaviors (Figures 2A, 3A).

A total of 316 animals were used in this study and, among them, 158 mice were subjected to chronic restraint stress/control, and 158 mice were subjected to cohabitation with a partner in stressful/control conditions. However, considering the losses of animals that did not reach the target structure related to stereotactic surgery from experiments 2 and 3, in the results item, 203 animals were considered in the statistical analysis.

### Histology

At the end of experiments 2 and 3, cagemate stress and cagemate control received intra-ACC or intra-amygdala infusion of 0.1 μL of 2% Evans blue according to the microinjection procedure previously described. After euthanasia in a carbon dioxide (CO_2_) chamber, the animal brains were removed and individually accommodated in containers containing formalin solution (10%). Subsequently, the brains were sectioned (50 μm) using a cryostat (Leica CM 1850, Leica Biosystems, Germany) at −20°C for histological analysis of the injection sites. Coronal sections were analyzed by microscopy (Olympus BX41, Olympus, Japan) and the visualization of the dispersion of Evans blue indicated the sites of the microinjections, according to the Paxinos and Franklin (2019) Mouse Brain Atlas. Data from animals with injection sites outside the amygdala and ACC were excluded from the study.

### Statistical analysis

All data were initially checked for homogeneity of variance (Levene’s test). For experiment 1 data were analyzed by Student’s *t*-test for independent samples. Data from experiments 2 and 3 were subjected to a two-factor ANOVA (cohabitation × treatment) analysis. For the weight gain, the data were analyzed using two-way ANOVA (stress exposure × cohabitation). When ANOVA analyses were statistically significant, Duncan’s *post-hoc* test was applied for the among-group comparisons. Results of statistical tests with a *p*-value ≤ 0.05 were considered significant.

## Results

### Body weight gain

In experiment 1, the two-way ANOVA [factor 1: stress exposure (control/stress) and factor 2: cohabitation (direct/indirect)] indicated significant differences for stress exposure [*F*_(1_,_26)_ = 45.70, *p* < 0.05], cohabitation factor [*F*_(1_,_26)_ = 42.65, *p* < 0.05], and for stress exposure vs. cohabitation interaction factors [*F*_(1_,_26)_ = 44.16, *p* < 0.05]. In experiment 2, the two-way ANOVA indicated significant differences for stress exposure [*F*_(1_,_48)_ = 23.51, *p* < 0.05] and for stress exposure vs. cohabitation interaction factors [*F*_(1_,_48)_ = 41.29, *p* < 0.05], without revealing significant differences for cohabitation factor [*F*_(1_,_48)_ = 1.97, *p* > 0.05]. In experiment 3, the two-way ANOVA indicated significant differences for stress exposure [*F*_(1_,_52)_ = 12.75, *p* < 0.05], and for stress exposure vs. cohabitation interaction factors [*F*_(1_,_52)_ = 14.70, *p* < 0.05], without revealing significant differences for cohabitation factor [*F*_(1_,_52)_ = 0.42, *p* > 0.05]. Duncan’s *post-hoc* test showed that in all experiments mice subjected to restraint stress gained less weight compared to the control group. Moreover, cagemate stress displayed greater weight gain compared to the stress group. Even more, cagemate control of experiments 2 and 3, that underwent stereotaxic surgery gained less weight compared to the respective control group (Table 1).

**TABLE 1:** Body weight gain during 14 days of restraint stress.

Experimental groups	Subject	1st stress day	14th stress day	Weight gain
Experiment 1 Immunofluorescence	Control	35.4 ± 0.5	43.4 ± 0.5	7.9 ± 0.2
	Cagemate control	36.1 ± 1.1	44.0 ± 1.1	7.9 ± 0.4
	Stress	34.8 ± 0.6	38.4 ± 0.7	3.6 ± 0.3**^#^**
	Cagemate stress	33.7 ± 0.7	41.6 ± 0.7	7.9 ± 0.3*
Experiment 2 ACC inactivation	Control	36.5 ± 1.0	44.7 ± 1.1	8.2 ± 0.3
	Cagemate control	34.6 ± 3.5	39.9 ± 4.1	5.6 ± 0.7**^#^**
	Stress	34.6 ± 1.3	36.4 ± 1.5	1.8 ± 0.6**^#^**
	Cagemate stress	37.2 ± 4.1	35.5 ± 4.7	6.2 ± 0.5*
Experiment 3 Amy inactivation	Control	40.1 ± 0.9	48.6 ± 1.0	8.4 ± 0.7
	Cagemate control	38.8 ± 1.3	43.8 ± 1.7	5.0 ± 0.8**^#^**
	Stress	37.6 ± 1.3	40.3 ± 1.1	2.8 ± 0.7**^#^**
	Cagemate stress	39.2 ± 1.2	44.4 ± 1.5	5.2 ± 0.7*

Data are presented as mean ± SEM. #*p* < 0.05 compared to the control. *p < 0.05 compared to the stress. ACC, anterior cingulate cortex; Amy, amygdala.

### Experiment 1. Living with mice subjected to chronic restraint stress induces a decrease of FosB labeling in the ACC, but not in the amygdala

Figure 1 represents the activation pattern through the FosB labeling in ACC [cagemate control (*n* = 7) and cagemate stress (*n* = 6)] and amygdala [cagemate control (*n* = 6) and cagemate stress (*n* = 6)] in mice that cohabited in pairs subjected to chronic stress as assessed by the immunofluorescence assay. For this experiment, 26 animals were used: 13 control and stress and 13 cagemate control and cagemate stress (Table 2).

**TABLE 2:** Sample sizes for each experimental group.

Experimental groups	Treatment	Condition			
		**Control**	**Stress**	**Cagemate control**	**Cagemate stress**
Experiment 1	FosB expression ACC and amygdala	7	6	7	6
Experiment 2	Saline	11	12	11	12
	CoCl_2_	11	13	11	13
Experiment 3	Saline	9	9	9	9
	CoCl_2_	10	14	10	14

The numbers represent the sample size used per group. Drug dose are represented in nmol/0.1 μl. Saline groups received a volume of 0.1 μl per hemisphere. The control and stress animals did not receive pharmacological treatment.

For ACC data, the Student’s *t*-test revealed a significant difference [*t*_(11)_ = 3.18; *p* < 0.05], showing that cagemate stress exhibited lower FosB-labeled immunofluorescence than those cagemate controls (Figure 1D). However, for amygdala data, the Student’s *t*-test revealed no significant difference [*t*_(10)_ = 0.38; *p* > 0.05], indicating a lack of effect of cohabitation with mice subjected to chronic restraint stress on FosB-labeled immunofluorescence in this forebrain area (Figure 1G).

### Experiment 2. Anxiogenesis induced by living in pairs with mice subjected to chronic restraint stress is reversed by ACC chemical inactivation

#### Histological analysis

The histological analysis confirmed that 47 mice had accurate microinjection bilaterally into the ACC [control/saline (*n* = 11); control/CoCl_2_ (*n* = 11); stress/saline (*n* = 12); stress/CoCl_2_ (*n* = 13)] (Figures 2B, C). For this experiment, 94 animals were used: 47 control and stress and 47 cagemate control and cagemate stress (Table 2).

#### ACC inactivation

Figures 2B, C represent a schematic diagram and a representative photomicrograph, respectively, of the microinfusion sites within the ACC. Two-way ANOVA (cohabitation × treatment) for %OE did not reveal significant effects for cohabitation [*F*_(1_,_43)_ = 1.62; *p* > 0.05] and treatment [*F*_(1_,_43)_ = 2.49; *p* > 0.05] factors, but indicated difference in cohabitation vs. treatment interaction [*F*_(1_,_43)_ = 9.54; *p* < 0.05] (Figure 2D). For %OT, two-way ANOVA revealed significant effects for cohabitation × treatment interaction [*F*_(1_,_43)_ = 8.52; *p* < 0.05], but not for cohabitation [*F*_(1_,_43)_ = 3.44; *p* > 0.05] and treatment [*F*_(1_,_43)_ = 0.02; *p* > 0.05] factors (Figure 2E). Two-way ANOVA did not reveal any effect for closed arm entries [cohabitation: *F*_(1_,_43)_ = 0.35; *p* > 0.05; treatment: *F*_(1_,_43)_ = 2.78; *p* > 0.05 and interaction: *F*_(1_,_43)_ = 1.68; *p* > 0.05] (Figure 2F). Duncan’s *post-hoc* test revealed a decrease in %OE (*p* < 0.05) and %OT (*p* < 0.05) in the cagemate stress-saline group when compared to the cagemate control-saline group. Furthermore, the treatment with CoCl_2_ (1 mM/0.1 μL) reversed the anxiogenic effect of cohabitation, through enhanced %OE (*p* < 0.05) and the %OT (*p* < 0.05) in cagemate stress compared to the respective cagemate stress-saline.

#### Complementary measures

A summary of the two-way ANOVA for complementary measures is shown in Table 3. Duncan’s *post-hoc* test revealed that cagemate stress-saline increased the percentage of protected head-dipping (*p* < 0.05) and percentage of protected SAP (*p* < 0.05) but decreased total head-dipping (*p* < 0.05) compared to cagemate control-saline. Intra-ACC microinjection of CoCl_2_ reversed cohabitation effects on the percentage of protected SAP (*p* < 0.05), protected head-dipping (*p* < 0.05), and total head-dipping (*p* < 0.05) compared to cagemate stress-saline.

**TABLE 3:** Effects of saline or CoCl_2_ (1 mM/0.1 μL) inactivation within the ACC (experiment 2) and amygdala (experiment 3) in anxiety induced by living with a conspecific submitted to chronic stress in mice exposed to EPM.

Experiment	Behavior	Cagemate control		Cagemate stress		Two way ANOVA
		**Saline**	**CoCl_2_**	**Saline**	**CoCl_2_**	
2	% Center time	19.7 ± 2.1	17.2 ± 2.8	18.7 ± 2.5	19.2 ± 2.2	^a^*F*_(1,43)_ = 0.02; *p* > 0.05
						^b^*F*_(1,43)_ = 0.16; *p* > 0.05
						^c^*F*_(1,43)_ = 0.35; *p* > 0.05
	Total SAP	45.2 ± 4.2	38.4 ± 2.9	47.1 ± 4.9	45.3 ± 3.2	^a^*F*_(1,43)_ = 1.26; *p* > 0.05
						^b^*F*_(1,43)_ = 1.20; *p* > 0.05
						^c^*F*_(1,43)_ = 0.41; *p* > 0.05
	% Protected SAP	52.5 ± 6.2	67.7 ± 7.6	78.7 ± 4.7**^#^**	60.7 ± 5.6	^a^*F*_(1,43)_ = 2.50; *p* > 0.05
						^b^*F*_(1,43)_ = 0.05; *p* > 0.05
						^c^*F*_(1,43)_ = 7.43; *p* < 0.05
	Total head-dipping	45.5 ± 4.4	36.1 ± 5.7	26.3 ± 3.2**^#^**	41.3 ± 3.2*	^a^*F*_(1,43)_ = 2.80; *p* > 0.05
						^b^*F*_(1,43)_ = 0.44; *p* > 0.05
						^c^*F*_(1,43)_ = 8.37; *p* < 0.05
	% Protected head-dipping	35.8 ± 6.9	51.8 ± 9.3	71.4 ± 7.9**^#^**	45.6 ± 6.6*	^a^*F*_(1,43)_ = 3.60; *p* > 0.05
						^b^*F*_(1,43)_ = 0.40; *p* > 0.05
						^c^*F*_(1,43)_ = 7.30; *p* < 0.05
3	% Center time	15.6 ± 2.9	17.3 ± 3.5	17.7 ± 2.8	15.8 ± 2.1	^a^*F*_(1,38)_ = 0.01; *p* > 0.05
						^b^*F*_(1,38)_ = 0.00; *p* > 0.05
						^c^*F*_(1,38)_ = 0.40; *p* > 0.05
	Total SAP	63.0 ± 6.8	46.6 ± 2.8*	50.2 ± 6.1	57.3 ± 2.7	^a^*F*_(1,38)_ = 0.04; *p* > 0.05
						^b^*F*_(1,38)_ = 1.00; *p* > 0.05
						^c^*F*_(1,38)_ = 6.46; *p* < 0.05
	% Protected SAP	50.9 ± 7.2	66.9 ± 7.1	79.5 ± 7.4**^#^**	78.4 ± 4.1	^a^*F*_(1,38)_ = 9.99; *p* < 0.05
						^b^*F*_(1,38)_ = 1.38; *p* > 0.05
						^c^*F*_(1,38)_ = 1.83; *p* > 0.05
	Total head-dipping	48.6 ± 3.4	46.4 ± 6.3	33.3 ± 4.2**^#^**	34.2 ± 3.3	^a^*F*_(1,38)_ = 9.44; *p* < 0.05
						^b^*F*_(1,38)_ = 0.02; *p* > 0.05
						^c^*F*_(1,38)_ = 0.12; *p* > 0.05
	% Protected head-dipping	35.3 ± 6.1	3.1 ± 0.4*	69.4 ± 9.1**^#^**	67.3 ± 5.7**^#^**	^a^*F*_(1,38)_ = 63.80; *p* < 0.05
						^b^*F*_(1,38)_ = 7.76; *p* < 0.05
						^c^*F*_(1,38)_ = 6.02; *p* < 0.05

The data represent mean ± SEM. #*p* < 0.05 compared to the respective cagemate control. *p < 0.05 compared to the respective cagemate saline. ^a^Cohabitation factor. ^b^Treatment factor. ^c^Interaction by cohabitation and treatment factors. CoCl**_2_**, cobalt chloride; EPM, elevated plus-maze; SAP, stretched attend postures.

### Experiment 3. Chemical inactivation of the amygdala did not change anxiety-like behaviors induced by living in pairs with mice subjected to chronic restraint stress

#### Histological analysis

The histological analysis confirmed that 42 mice had accurate microinjection bilaterally into the amygdala [control/saline (*n* = 9); control/CoCl_2_ (*n* = 10); stress/saline (*n* = 9); stress/CoCl_2_ (*n* = 14)] (Figures 3B, C). For this experiment, 84 animals were used: 42 control and stress and 42 cagemate control and cagemate stress (Table 2).

#### Amygdala inactivation

Figures 3B, C represent a schematic diagram and a representative photomicrograph, respectively, of the microinfusion sites within the amygdala. Two-way ANOVA (cohabitation × treatment) for %OE revealed significant effects for cohabitation [*F*_(1_,_38)_ = 16.12; *p* < 0.05], but not for treatment factor [*F*_(1_,_38)_ = 0.17; *p* > 0.05] and cohabitation vs. treatment interaction [*F*_(1_,_38)_ = 0.08; *p* > 0.05] (Figure 3D). For %OT, two-way ANOVA revealed significant effect for cohabitation [*F*_(1_,_38)_ = 16.17; *p* < 0.05], but not for treatment factor [*F*_(1_,_38)_ = 0.38; *p* > 0.05] and cohabitation vs. treatment interaction [*F*_(1_,_38)_ = 0.67; *p* > 0.05] (Figure 3E). Two-way ANOVA did not reveal any effect for closed arm entries [cohabitation: *F*_(1_,_38)_ = 0.62; *p* > 0.05; treatment: *F*_(1_,_38)_ = 0.73; *p* > 0.05 and interaction: *F*_(1_,_38)_ = 0.00; *p* > 0.05] (Figure 3F). Duncan’s *post-hoc* test revealed a decrease in %OE (*p* < 0.05) and the %OT (*p* < 0.05) in cagemate stress-saline when compared to cagemate control-saline. Furthermore, the treatment with CoCl_2_ (1 mM/0.1 μL) did not reverse the anxiogenic effect of cohabitation, as observed by the lack of effects on %OE (*p* > 0.05) and %OT (*p* > 0.05) in cagemate stress compared to the respective cagemate stress-saline.

#### Complementary measures

A summary of the two-way ANOVA analysis is shown in Table 3. Duncan’s *post-hoc* test revealed that cagemate stress-saline increased the percentage of protected head-dipping (*p* < 0.05) and percentage of protected SAP (*p* < 0.05), and a decrease in total head-dipping (*p* < 0.05). Intra-amygdala microinjection of CoCl_2_ reduced the percentage of protected head-dipping (*p* < 0.05) and total SAP (*p* < 0.05) only in cagemate stress-saline (see Table 3 for details).

## Discussion

The present study corroborates previous evidence wherein living with a cagemate in distressing conditions produces anxiogenic-like effects in the observer mouse exposed to the EPM (Carneiro de Oliveira et al., 2017). Furthermore, we found a drop-off in the FosB-labeled cells in the ACC in animals living with a stressed conspecific; however, the cohabitation with a partner in a stressful situation did not change FosB-labeled cells in the amygdala. In the same way, the temporary functional inactivation (induced by intra-ACC, but not intra-amygdala, injection of CoCl_2_), reversed the anxiogenic-like effect induced by cohabitation with a stressed mouse.

Our data also demonstrated that the 1-h/day for 14 consecutive days of restraint stress (stressed group) led to a decrease in body weight gain. Previous studies have associated weight loss as an effect of chronic variable stress (Prewitt and Herman, 1997; Cruz et al., 2016), chronic restraint stress (Ribeiro et al., 2018), and unpredictable mild stress (Zhu et al., 2014). In this context, we suggest that the impairment of body weight gain induced by restraint stress protocol is related to an increase in energy expenditure and a decrease in the intake of food during the days of chronic stress (Chotiwat and Harris, 2006; Carneiro de Oliveira et al., 2017). In light of this findings, it is well-established that restraint stress causes acute hyperthermia in rats (Harris et al., 2002, 2006; Mota-Rojas et al., 2021). Stress-induced hyperthermia is defined as an integral part of a physiological response, characterized by an increase in body temperature that is generated from threats to homeostasis caused by stressful stimuli (Lees et al., 2020). Prior findings indicate that temperature control is essential for survival (Song et al., 2016; Wang et al., 2019). Since throughout evolution, several adaptive mechanisms have occurred to face changes that the environment or its habitat may undergo (Morrison and Nakamura, 2011; Villanueva-García et al., 2021). Thus, despite the existence of an important activation of thermogenesis due to the consumption of brown adipose tissue (Harris et al., 2006), there is also an important thermogenesis of cardiac origin, such as increased blood pressure (Crestani, 2016), contributing to a decrease in the release of heat to the external environment. In line with previous reports, despite energy expenditure not being the focus of the study, we suggest that the lower weight gain observed in the stressed animal led to an increase in energy expenditure triggered mainly by the increase in body temperature. Even so, tail skin temperature could be used as an indirect measure of sympathetic-mediated cutaneous vasoconstriction (Benini et al., 2019) and would be a useful measure for future studies. Moreover, we have to highlight that cagemate control and cagemate stress displayed greater weight gain than stress-group, but less than the control group in both experiments 2 and 3. This discrepancy may be due to the surgery of cannulae implantation since it did not happen in the same groups from experiment 1.

The behavioral results shown in the present study also corroborate our previous findings reported by our research group showing that living with cagemates subjected to chronic restraint stress can induce anxiogenic-like behaviors in mice exposed to the EPM (Carneiro de Oliveira et al., 2017). These behavioral data suggest that mice can perceive their conspecifics under suffering, through emotional contagion. Corroborating this suggestion are previous studies showing enhanced anxiety-like behaviors in cagemates cohabitating with mice in distress conditions (Jeon et al., 2010; Baptista-de-Souza et al., 2015; Benassi-Cezar et al., 2020).

Based on the behavioral results that suggest an emotional contagion induced by restraint stress in mice, we evaluated the role of the ACC and amygdala activation patterns through FosB neuronal labeling in mice chronically exposed to a pair stressed (experiment 1). The cagemate stress group showed decreased FosB in the ACC without revealing any change of this neuromarker within the amygdala. We have recently demonstrated a decrease in the expression of FosB in the ACC of mice exposed to a conspecific undergoing chronic pain (Benassi-Cezar et al., 2020). It is important to highlight that in both studies, experiencing conspecific’s suffering promotes anxiogenic-like behaviors in EPM. Similar results demonstrated diminished c-Fos expression in ACC of rats exhibiting high anxiety behaviors (Salomé et al., 2004). Furthermore, a decrease of Fos-positive cells expression in the ACC and an increase in anxiety-like behaviors were also found in rats subjected to an animal model of posttraumatic stress disorder (Liu et al., 2019; Qu et al., 2021). These sets of evidence lead us to suggest that cohabitation with a cagemate in chronic pain or subjected to chronic stress induces a reduction in ACC neuronal activation. In this way, an interesting work reported that in addition to provoking anxiogenic-like behavior, chronic restraint stress promotes a reduction in ACC volume due to decreasing synaptic density (Misquitta et al., 2021).

The ACC has been deeply implicated in anxiety modulation responses in clinical and preclinical studies (Gross and Hen, 2004; Kim et al., 2011). In animal models, studies have showed the increase of activation patterns in the prefrontal areas (that includes the ACC) of stressed animals (Knapska et al., 2010; Burkett et al., 2016). Taking into account, we expected to find an increase (instead of a decrease) in FosB measures within ACC. However, it is relevant to highlight that these previous studies have used different stressor stimuli, rodent species, Fos gene targets, as well as reached distinct subareas. Generally, these procedural differences among the present work and those reported in previous studies might explain such contrasting results. In this context, we do not have a clear explanation for these results yet. However, considering that Jhang et al. (2018) showing predominantly GABAergic neuronal activation within the ACC of rats during an innate fear test and that FosB proteins accumulation occurs mainly in glutamatergic neurons (Vialou et al., 2010, 2015), we parsimoniously suggest that the diminished FosB immune labeling observed in ACC might be related to the activation of local GABAergic neurons. Taking this account, the accentuation of GABAergic neurotransmission would inhibit the glutamatergic neurotransmission in the modulation of harmful situation in mice under the influence of strong emotional conditions. This is an important hypothesis that needs to be addressed in the future study, through double-labeling techniques. Additionally, although a decrease of FosB-positive cells expression in the ACC has already been described in restrained mice (Liu et al., 2019), the novelty revealed in the present study was a decrease in activation patterns in the ACC in cagemates cohabitating with chronic restraint stress animals, showing not only a simple psychological stress, but a complex process that requires perception of the aversive condition, recognition of negative emotional state from another, and engagement in relieving conspecific distress (Carneiro de Oliveira et al., 2022).

In experiment 2, we evaluated the effects of ACC inactivation on anxiety-like behavior in cagemates living with chronically stressed mice. Intra-ACC CoCl_2_ microinjection reversed the anxiogenic behaviors displayed by cagemate stress compared to the respective cagemate stress saline group. This result corroborates our previous findings showing that inhibition of ACC through local injection of CoCl_2_ reversed the increased anxiety-like behavior induced by the empathy for pain model in mice (Benassi-Cezar et al., 2020). Besides reversing the conventional measures of anxiety (i.e., %OE and %OT), ACC CoCl_2_ inactivation also impaired the anxiogenesis as assessed by the complementary measures. Intra-ACC CoCl_2_ prevented the effects of living with a cagemate suffering chronic stress on the percentage of protected SAP, protected head-dipping, and total head-dipping. These defensive exploratory behaviors (complementary measures) have been used as subtle parameters of anxiety (Rodgers and Johnson, 1995; Sorregotti et al., 2013). Notably, the effects of CoCl_2_ were selective on anxiety measures because this synaptic inhibitor did not change the frequency of closed arm entries, a valid index of locomotor activity (Rodgers and Johnson, 1995; Sorregotti et al., 2013). In this sense, the results shown here corroborate previous studies showing that ACC inactivation leads to a reduction of anxiety-related behaviors. For instance, Jeon et al. (2010) demonstrated that mice subjected to ACC inactivation with local injection of lidocaine (sodium channel blocker) showed impairment of observational fear learning characterized by reduction of freezing when observing a conspecific receiving a footshock. Conversely, in a recent study, socially isolated mice exhibited augmented anxiety behavior in the EPM test after ACC injection of muscimol, a GABA_A_ receptor agonist (Chaibi et al., 2021). A plausible explanation for these opposite results rises from the mechanism of CoCl_2_ and muscimol. The divalent CoCl_2_ decreases by competing for calcium influx in the presynaptic terminal, thereby preventing calcium uptake at the presynaptic terminal and subsequent release of a synaptic neurotransmitter (Kretz, 1984). The microinjection of CoCl_2_ is an advantageous pharmacological tool to study the overall influence of a central area when compared to other neuronal inhibitors (Brasil et al., 2020). In addition, the dose of CoCl_2_ (1 mM) used in the present study is effective in the blockade of synaptic transmissions and has been extensively used to study the involvement of brain areas in the neural circuitry of emotional responses related to empathy (e.g., Benassi-Cezar et al., 2020; Tavares et al., 2021, 2022).

Although the role of the neurotransmission signaling within the ACC in the anxiety-like behavior induced by living with a conspecific subjected to chronic restraint stress was not in the scope of this study, GABA and glutamate are strong candidates in this mediation. In the rat ACC, GABA_A_ inhibitory receptors and AMPA glutamatergic receptors were present in significantly higher concentrations (Bozkurt et al., 2005; Waldvogel et al., 2017). If so, we suggest that the inhibition of the ACC (with local injection of CoCl_2_) could have prevented the release of GABA in this structure, which in turn could lead to an increase of glutamate release reducing the anxiogenesis induced by living with a cagemate suffering chronic stress. In this sense, as muscimol stimulates the GABA_A_ receptor and consequently inhibits post-synaptic glutamate neurons, inducing anxiogenic-like effects (Chaibi et al., 2021). On the other hand it is important to highlight that microinjections of a channel inhibitor into the ACC was also able to reduce the increase of anxiety triggered by pain in a model of chronic pain (Koga et al., 2015) representing that in different animals protocols the glutamatergic neurotransmission in the ACC could modulate anxiety responses in contrasting ways (Koga et al., 2015; Roshan-Milani et al., 2021).

Regarding the expression of FosB in the amygdala, the results showed a lack of effects in cagemate-stress compared to cagemate control. Similar results have been reported by our group, where we demonstrated that cohabitation with a pair subjected to chronic pain did not change the FosB-labeled cells in the amygdaloid complex compared to the sham group (Tavares et al., 2021). In contrast, Kormos et al. (2016) found an increase in FosB measures within the amygdala in mice exposed to variable chronic stress. Perhaps some methodological variance may have influenced these discrepancies regarding the role of the amygdala in the mediation of stress-related situations, i.e., while Kormos et al. (2016) have exposed the animals to chronic variable stress, the current work investigated the conspecific that co-housed with a pair exposed to restraint stress.

Amygdala inactivation with local injection of CoCl_2_ did not prevent the anxiety-like behavior in mice housed with stressed animals. Specifically, cagemate stressed animals that received saline intra-amygdala explored less the open arms of the EPM than cagemate control, an effect that was not changed by the amygdala inhibition, suggesting lack of amygdala role in empathy-related and/or social behaviors. Similarly, bilateral amygdala lesions through kainic acid failed to affect empathic response to a familiar conspecific in pain (Li et al., 2014). In addition, Sorregotti et al. (2018) demonstrated that amygdala inactivation with CoCl_2_ did not alter defensive behaviors exhibited by mice exposed to an open EPM.

On the other hand, it has been well-known that the amygdala is a brain structure that coordinates emotional responses involving endocrine and neurochemical mechanisms associated with stress (e.g., Pêgo et al., 2009). This structure comprises several subnuclei, making complex connections with other limbic areas *via* distinct pathways and playing various roles in response to stress (Sah et al., 2003; Zhang et al., 2021). Thus, due to technical difficulty to reach specific amygdala subnuclei through stereotaxic surgery in mice, we suggested that the lack of effects in this experiment might be a consequence of the specific role of each subnucleus in modulating emotional responses (Salomé et al., 2004; Horovitz et al., 2017). Accordingly, taking into account that both FosB labeling and functional inactivation approaches were assessed in the structure as a whole, the present results did not clarify the involvement of the amygdala in the anxiety response induced by emotional contagion. In light of these findings, previous evidence has already reported the opposite functions of amygdaloid subnuclei in the control of emotional responses (Manassero et al., 2018). It seems reasonable to suggest that these inconsistencies shown here need to be investigated in further studies through pharmacological and molecular manipulations in specific amygdala subnuclei to clarify the role of these independent circuits in empathy animal models.

Moreover, it is not unreasonable to suggest that the lack of effects of amygdala FosB labeling and inactivation concerns the maturation of the amygdala related to social behaviors. The amygdala’s excitatory and inhibitory circuits mature during adolescence, and the prefrontal cortex inputs to the basolateral nucleus (BLA) gain a more remarkable ability to recruit intra-BLA inhibitory networks by adulthood (Ferrara et al., 2021). Studies have shown changes in amygdala structure and function over development, and that exposure to stress disrupts the normal development of the amygdala. The volume of amygdala, especially the BLA in rats, increases by 113% from birth to 3-week old with an additional 33% increased by 7 months of age (Chareyron et al., 2012). This description is justified because, in all the experiments, the pairs begin to live together with 21 days of life (PND 21) considered adolescent animals.

Adolescents are highly vulnerable to stressors and the consequences of stressors on social behaviors (Ferrara et al., 2021), being a critical period for brain development, characterized by neuro-anatomical rearrangements (Zhang and Rosenkranz, 2012). Following repeated stress exposure, *in vivo* BLA neuronal firing activity is elevated in adults, while in adolescents increase, the number of active neurons encountered (Zhang and Rosenkranz, 2012). Also, chronic restraint stress increases neuronal membrane excitability in adolescents throughout the BLA (Hetzel and Rosenkranz, 2014), similar to what is seen in the BLA after post-weaning social isolation (Rau et al., 2015). These identify adolescence as a period of raised sensitivity to social disruptions, particularly social stressors, compared to adults (Vidal et al., 2007). Together, this frames a potential effect, wherein stressors can particularly influence adolescence, disrupting social behaviors and conducting further impairments. Taken in this context, along with previous literature, we suggest that although the animals used in this study were at PND 50 on the test day, a period considered as late adolescence (Vidal et al., 2007; Ferrara et al., 2021), we cannot rule out that part a result of immature amygdala function. However, further studies are necessary to clarify this hypothesis.

In summary, our results imply that living with a conspecific subjected to chronic restraint stress decreases FosB labeling in the ACC without changing FosB labeling cells in the amygdala. Synaptic inhibition attenuated and did not alter the anxiogenic like-behavior after CoCl_2_ into the ACC and the amygdala, respectively, in cagemate stress. These results suggest that the ACC (but not the amygdala) plays an essential role in modulating anxiety induced by emotional contagion in mice. Nevertheless, it is necessary to mention that this effect was observed in familiar mice, that cohabited together for 28 days. Since the interaction with an unfamiliar conspecific induces stress response in mice (Martin et al., 2015; Meyza et al., 2017) the present results suggested that familiarity between conspecifics in our study is substantial and well-established by strong evidence showing the effect of emotional contagion in distress conditions. In line with this reasoning, considering that psychosocial stress-induced anxiogenic responses and, consequently, a decrease in social interaction (DiSabato et al., 2021; Carneiro de Oliveira et al., 2022) we cannot disregard that the results we found could be also consequences of the social interactions attenuation. However, additional studies with isolated animals are needed to fully understand how this factor could influence the anxiety responses. Therefore, for future studies it is important to investigate the role of other limbic structures (e.g., nucleus accumbens and paraventricular nucleus of hypothalamus) in the modulation of anxiety-like behaviors induced by empathy.

## Data availability statement

The raw data supporting the conclusions of this article will be made available by the authors without undue reservation.

## Ethics statement

The animal study was reviewed and approved by Federal University of São Carlos Ethics Committee on the Use of Animals (CEUA-UFSCar 7821030418) which complies with Brazilian and international guidelines for animal use and welfare.

## Author contributions

LS, LT, and IC carried out the experiments, data analysis, and drafting of the manuscript. LT, DB-d-S, and PC edited and revised the manuscript. RN-d-S critically revised the manuscript. AC-d-S conceived the study, supervised the experiments, performed the manuscript, wrote the supervision, and approved the final version to be published. All authors contributed to the article and approved the submitted version.
